# Pieces of the 3D puzzle: Identification of genes underlying rice canopy architecture

**DOI:** 10.1093/plphys/kiac474

**Published:** 2022-10-13

**Authors:** Alexandra J Burgess, Mateusz Majda

**Affiliations:** Agriculture and Environmental Sciences, School of Biosciences, University of Nottingham, Loughborough, UK; Department of Plant Molecular Biology, University of Lausanne, Lausanne, Switzerland

Canopy architecture, the arrangement of above-ground plant material in three-dimensional (3D) space, is critical in determining the interception of key resources including light and therefore underlies photosynthetic performance (Murchie and Burgess, 2022). Architectural traits determine the interception and use of resources, including light, water, nutrients, and gases, as well as influence the prevalence of pests, diseases, and pollinators. The structure of modern crop cultivars is a result of the genetics of the progenitors combined with ecological diversification and local cultural pressures. The genetic changes associated with domestication and canopy architecture of modern varieties have been mainly studied for members of the grass family. A common set of targeted traits includes loss of seed shattering, dormancy, branch angle or pattern, and internode elongation; collectively referred to as the “domestication syndrome” (Teichmann and Muhr, 2015).

Domestication in cereals is largely associated with changes in the pattern and timing of branching, which influences both vegetative and inflorescent structures (Doust, 2007). As such, changes in signaling pathways of phytohormones, including gibberellins, auxin, and cytokinins, provide key control mechanisms.

Compared to wild grass relatives, domesticated cereals have reduced levels of vegetative branching plus a reduced growth stature, and the genetic regions and environmental variables contributing to this have been identified for several species, including rice (*Oryza sativa*), millet (*Setaria sp.*), and maize (*Zea mays*) (Li et al., 2003; Duggan et al., 2005; Ishikawa et al., 2005; Doust and Kellogg, 2006). In addition to a reduction in tillering (the generation of a specialized branch or tiller), the angle between the tiller and the culm (the main or mother stem) is important for rice cultivation, with a more acute angle, and thus upright structure, permitting dense cultivation and a more horizontal angle facilitating weed suppression (Haefele et al., 2004; Wang and Li, 2006). Some genes associated with the orientation of branching structure have been identified, including *TILLER ANGLE CONTROL1* (*TAC1*) and *PROSTRATE GROWTH1* (*PROG1*), which controls the erect growth habit in Asian rice (Yu et al., 2007). However, the regulatory mechanisms controlling architecture are still relatively unknown.

In this issue of the *Plant Physiology*, Zhang et al. (2022) and Huang et al. (2022) present two regulatory components controlling plant architecture in rice. Zhang et al. (2022) describe the discovery of a recessive mutant presenting dwarf and reduced tillering phenotypes (*drt1*), which they trace to a single-point mutation in *DRT1*. This gene encodes the Class I protein, formin-like13 (OsFH13). The authors performed a binding assay and showed that the FH1-FH2 fragment of DRT1 binds to actin filaments and promotes actin polymerization in a concentration-dependent manner. These findings demonstrate that mutations affecting the cytoskeleton can have profound effects on plant architecture. Furthermore, through interaction with blue-light receptor phototropin2 (OsPHOT2), DRT1 controls light-mediated chloroplast relocation. The *drt1 phot2* double mutant showed lower chloroplast response to light stimulation than the single *phot2* mutant, confirming that DRT1 and PHOT2 are involved in the same pathway mediating light response. As such, DRT1 is an integral component of plant morphology and chloroplast relocation through modulation to the actin-associated cytoskeletal network.

In contrast, Huang et al. (2022) identify the role of cytokinin accumulation in the control of lamina joint development and leaf angle regulation. Cytokinin controls cell division, differentiation, and apical dominance. A proper balance between accumulation and degradation is essential to maintain suitable phytohormone levels. Cytokinins are degraded by oxidases (CKXs) that modify their local distribution and amount in plant tissues (Chen et al., 2020). Huang et al. (2022) discovered that through action of CYTOKININ OXIDASE/DEHYDROGENASE3 (OsCKX3), the altered cytokinin distribution led to an asymmetric proliferation of cells and vascular bundles in the lamina joint, resulting in negative regulation to leaf angle. Thus, knockout mutants exhibited smaller leaf angles, and overexpression mutants exhibited larger leaf angles. Field-grown *osckx3* mutants also displayed smaller leaf angles and increased primary branch number, grain size, grain weight, and flag leaf size.

These two genes join a list of previously identified targets controlling architecture in rice (Figure 1). While it is likely that other genes remain to be identified and their mode of action elucidated, findings presented in this issue bring us one step closer to determining the interplay between genotype and phenotype. As such, these findings offer the possibility for improving plant performance under diverse conditions, for example, through adaptation to different light environments by altered DRT1-mediated chloroplast relocation or by enhancing leaf erectness through changes in cytokinin homeostasis. Similarly, while both genes have been identified in rice, it is likely that homologs or orthologs are also present in other grass species, including key cereal crops, or even in broadleaf species.

**Figure 1:**
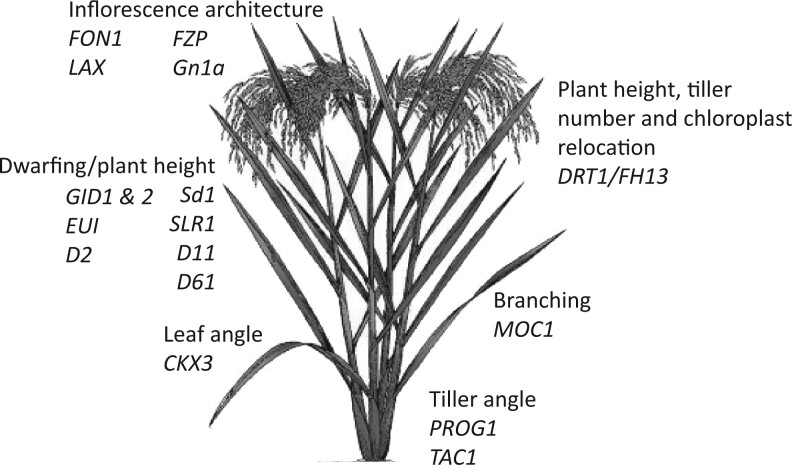
Overview of genes controlling shoot architecture in rice. Inflorescence architecture is known to be determined by *FLORAL ORGAN NUMBER1 (FON1), LAX PANICLE (LAX), FRIZZY PANICLE (FRZ)*, and *Gn1a*, which encodes the cytokinin oxidase/dehydrogenase OsCKX2. Plant height is determined by genes including *GIBBERELLIN-INSENSITIVE DWARF1* and *2 (GID1&2), ELONGATED UPPERMOST INTERNODE (EUI), ebisu dwarf (D2)* and *dwarf61 (D61), Semidwarf1 (Sd1), Slender rice1 (SLR1), Dwarf11 (D11)*, and *dwarf and reduced tillering1 (DRT1)*, which encodes the Class I formin protein OsFH13*.* This latter gene is also known to control tiller number and chloroplast relocation through interaction with the blue-light photoreceptor. Branching or tillering is controlled by *Monoculm1 (MOC1)*, while tiller angle is determined by *PROSTRATE GROWTH1 (PROG1)* and *Tiller Angle Control1 (TAC1).* Finally, leaf angle is determined by the cytokinin oxidase/dehydrogenase *CKX3.* Further details can be found in (Wang and Li, 2006; Yu et al., 2007; Huang et al., 2022; Zhang et al., 2022).

## Funding

A.J.B. is supported by the Leverhulme Trust as an Early Career Fellow (ECF-2020-224).

*Conflict of interest statement*: The authors declare no conflict of interest.
